# Concomitant Coronary Artery Disease in Identical Twins: Case Report and Systematic Literature Review

**DOI:** 10.3390/jcm12175742

**Published:** 2023-09-03

**Authors:** Odysseas Kamzolas, Andreas S. Papazoglou, Eleftherios Gemousakakis, Dimitrios V. Moysidis, Kοnstantinos G. Kyriakoulis, Emmanouil S. Brilakis, Anastasios Milkas

**Affiliations:** 1 401 General Military Hospital of Athens, 11525 Athens, Greece; odyskam17@hotmail.com (O.K.); leogemou@gmail.com (E.G.); 2 Athens Naval Hospital, 11521 Athens, Greece; anpapazoglou@yahoo.com (A.S.P.); konkyriakoulis@gmail.com (K.G.K.); 3 424 General Military Hospital of Thessaloniki, 56429 Thessaloniki, Greece; dimoysidis@gmail.com; 4 Center for Coronary Artery Disease, Minneapolis Heart Institute and Minneapolis Heart Institute Foundation, Abbott Northwestern, Minneapolis, MN 55407, USA; esbrilakis@gmail.com

**Keywords:** coronary artery disease, epigenetic, genetic, meta-summary, twin studies

## Abstract

Coronary artery disease (CAD) is multifactorial and strongly affected by genetic, epigenetic and environmental factors. Several studies have reported development of concomitant CAD in identical twins. We report a case in which a pair of Caucasian male monozygotic twins presented almost concomitantly with acute coronary syndrome (ACS) and had concordant coronary anatomy and identical site of occlusion. We performed a systematic literature review of PubMed, Web Of Science and Scopus databases from inception until 28 February 2023 of case reports/case series reporting the concomitant development of CAD in monozygotic twins. We found 25 eligible case reports with a total of 31 monozygotic twin pairs (including the case from our center) suffering from CAD and presenting (most of them simultaneously) with ACS (mean age of presentation: 45 ± 12 years, males: 81%). Coronary angiograms demonstrated lesion and anatomy concordance in 77% and 79% of the twin pairs, respectively. Screening for disease-related genetic mutations was performed in six twin pairs leading to the identification of five CAD-related genetic polymorphisms. This is the first systematic literature review of studies reporting identical twin pairs suffering from CAD. In summary, there is high concordance of coronary anatomy and clinical presentation between monozygotic twins. Future monozygotic twin studies—unbiased by age effects—can provide insights into CAD heritability being able to disentangle the traditional dyad of genetic and environmental factors and investigate the within-pair epigenetic drift.

## 1. Introduction

Coronary artery disease (CAD) is the leading cause of morbidity and mortality worldwide [1]. Its prevalence is expected to rise within the next few years because of the increasing incidence of traditional cardiovascular risk factors (such as smoking, metabolic syndrome and decreased physical activity) and non-traditional risk factors under investigation (such as noise and air pollution) [2]. In addition to those environmental factors predisposing to CAD, a heritable component has also been well-established [3]. Heritability is a measure of how well genomic differences account for phenotypic trait discordance among separate individuals [3]. Genome-wide association studies (GWAS) have transformed our understanding of many heritable traits [2]. Currently, research on CAD genetics and genomics is burgeoning and tremendous success has been achieved in elucidating potential causal genetic loci and responsible mutations (single nucleotide polymorphisms, SNPs) that can significantly modulate the risk for CAD occurrence, including the genes responsible for the expression of the atherogenic lipoprotein(a), apolipoprotein E or for the defects in cholesterol metabolism which might even have a causal role for CAD [2,4,5,6,7].

Twin studies offer an opportunity to assess the influence of genetic, epigenetic and environmental factors, because twins share the same intrauterine conditions, birth date and are exposed to similar environmental conditions in early stages of life [8]. Monozygotic pairs have the same chromosomal DNA sequence, except for very small errors of DNA replication, while dizygotic pairs share many maternal and obstetric factors; however, the likelihood of sharing specific genetic variations is the same as that of non-twin siblings [8]. Therefore, twin studies play a pivotal role in the discovery of the genetic basis and risk factors of CAD [8,9,10].

In this manuscript, we describe a case of a monozygotic twin pair with concordant CAD presentation and a systematic review and meta-summary of similar published case reports.

## 2. Materials and Methods

### 2.1. Case Report Presentation

We report a case of concomitant CAD in identical twins treated in Athens Naval Hospital (Athens, Greece) with anonymized photos of the lesions from the catheterization laboratory and next-generation whole-exome sequencing analyses.

### 2.2. Systematic Review Design, Search Strategy and Study Selection

This systematic review and meta-summary of case reports was performed according to the protocol proposed by Nambiena et al. since the available systematic review guidelines (PRISMA, PRISMA-P, and Navigation Guide) remove from consideration any case reports and case series (as human subjects’ research with no control group), due to challenging assessments of their internal validity [11]. The framework and methodology observed for the current meta-summary of case reports has been registered a priori in the Open Science Framework platform (https://doi.org/10.17605/OSF.IO/TWXKP, accessed on 1 August 2023).

A comprehensive electronic search of PubMed, Web of Science and Scopus databases was performed independently by two investigators (OK and EG) from inception until Feb 28, 2023 to identify case reports or case series reporting concomitant CAD in monozygotic twin pairs. Our search included the following subject terms: “coronary artery disease”, “coronary syndrome” and “twins”.

We included original peer-reviewed case reports or case series describing reports of concomitant CAD in identical twins. Reports of concomitant CAD in fraternal (dizygotic) twins were excluded. Articles were screened by title, abstract and full text. The references of all included articles were searched to identify additional studies. Disagreements were resolved after consensus with a senior author (KGK).

### 2.3. Data Extraction

For each report, the following data were extracted by two researchers (OΚ and EG) where available: gender, age, predisposing cardiovascular risk factors (family history of CAD, dyslipidemia, hypertension, diabetes mellitus, obesity and smoking history), investigated and/or identified disease-related genes, baseline laboratory workup, presenting condition and symptoms, therapy (PCI/CABG), coronary anatomy concordance, diseased vessel location and lesion concordance.

### 2.4. Assessment of the Methodological Quality of Case Reports and Case Series

The methodological quality of the included case reports and case series was evaluated according to Murad et al. [12] in each of the following domains: selection, ascertainment, causality and reporting. Each study was rated with a maximum score of 5 stars, according to the corresponding questions in each domain.

### 2.5. Statistical Analysis and Data Synthesis

Data extracted and tabulated were further synthesized to derive pooled estimates of variables of interest. Results were expressed as mean ± SD for continuous variables and as n (%) for categorical variables. Concordance regarding comorbidities, coronary artery anatomy and angiographic lesions was assessed as the percentage of twins presenting with similar characteristics. Each percentage was calculated according to the number of studies with available data (e.g., the percentage of studies with missing data was not taken into consideration when calculating the percentage of twin pairs with concordant anatomy).

## 3. Results

### 3.1. Case Report

Two 47-year-old monozygotic male twins presented to a tertiary hospital suffering from ACS (myocardial infarction and unstable angina, respectively) with a 3-month difference in their presentation. They were both smokers without any other modifiable risk factor while family history of CAD was unknown. An interesting fact arose that they had been separated shortly after their birth and illegally adopted by different parents indicating the absence of common environmental factors shared by the twin pair. Coronary angiograms demonstrated concordant coronary anatomy and obstructive CAD in two major epicardial coronary vessels (left anterior descending artery (LAD) and right coronary artery (RCA)) with nearly identical lesions. Both twins had high lipoprotein(a) levels (Lp(a), 170 and 120 mg/dL, respectively), mildly elevated triglyceride (TRG, 130 and 110 mg/dL) and low-density lipoprotein cholesterol (LDL-C, 90 and 105 mg/dL) levels.

Aiming to further investigate the almost identical CAD observed in the two twins, next-generation whole-exome sequencing analyses were performed investigating the presence of any of the CAD-related genes mentioned in Supplementary Figure S1; however, no CAD -associated or lp(a)-related mutation could be identified.

### 3.2. Meta-Summary of Case Reports

**Figure 1:**
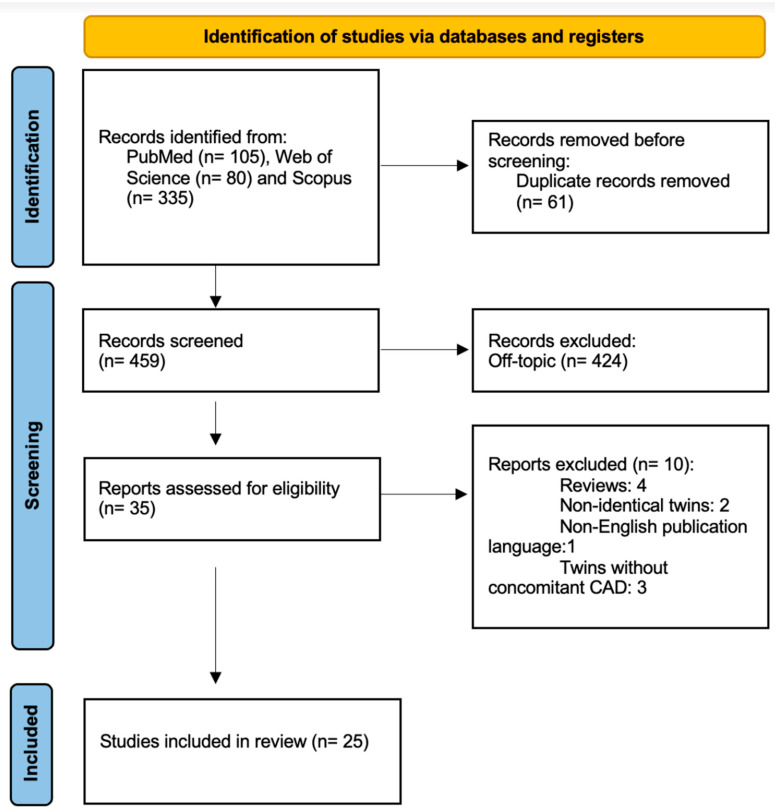
PRISMA flow diagram of the systematic review.

The literature search yielded initially a total of 560 results, of which 485 were excluded during screening as they did not report the relevant data or were duplicates (Figure 1). A total of 25 case reports or case series [13,14,15,16,17,18,19,20,21,22,23,24,25,26,27,28,29,30,31,32,33,34,35,36,37] were included with a total sample size of 31 pairs of identical twins, including the pair of our case report (not previously published).

### 3.3. Patient Baseline Characteristics

Mean age of the studied twins was 45 ± 12 years, with a male to female ratio of almost 4:1. All of them suffered concomitantly from CAD. In all included cases, at least one sibling presented with an acute coronary syndrome (ACS) and in 25/31 pairs, both twins had ACS. The most common presenting condition was unstable angina (44%), followed by acute myocardial infarction (AMI; 40%).

At the time of the ACS, 38 (61%) twins had chest pain and 6 (10%) had dyspnea. Other symptoms such as nausea or excessive sweating were encountered less frequently. There was no specific symptom recorded for 18 twins (29%).

Dyslipidemia was the major risk factor in most twins (60%), while smoking—or ex smoking—was reported in 32/62 twins (52%). Twenty-one twins (34%) had hypertension and ten twins (16%) had diabetes or pre-diabetes. Seven twins were obese (11%). Other risk factors for CAD such as alcohol/drug abuse or hypothyroidism were rare. A family history of premature CAD was present in 16/20 (80%) twin pairs (no data were reported for the remaining 11 pairs (35%)).

Total cholesterol levels were measured in eight twin pairs with a mean value of 256 ± 44 mg/dL, while the mean LDL-C value was 134 ± 76 mg/dL in five pairs. Triglyceride levels were recorded in 19 twins with a mean value of 189 ± 108 mg/dL, and Lp(a) levels were measured in 7 twins with a mean value of 207 ± 73 mg/dL. The average within-pair total cholesterol, LDL-C and TRG difference were 43, 27 and 67 mg/dL, respectively. The detailed baseline characteristics of the studied twin pairs are presented in Table 1 and Supplementary Table S1.

### 3.4. Procedural Characteristics

**Figure 2:**
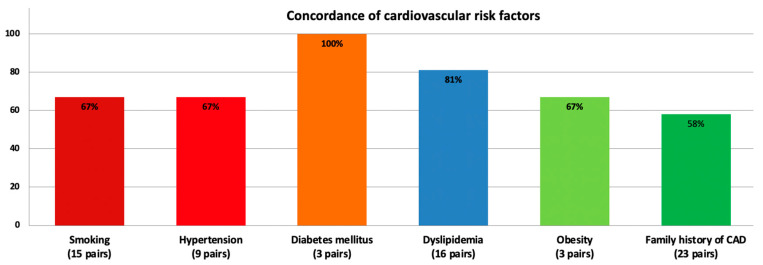
Concordance of cardiovascular risk factors within the twin pairs under investigation, with lesion concordance and available data for both twins (23 of the total 31 pairs).

Nineteen twin pairs (19/24; 79%) had concordant anatomy in their coronary vessels, while five pairs (21%) had anatomy discordance. Coronary vessel anatomy was not recorded in seven pairs. Coronary angiography showed identical lesions in 24 twin pairs (77%). The concordance of cardiovascular risk factors within the 24 twin pairs with lesion concordance is presented in Figure 2.

Coronary lesions were more common in the RCA (55%) followed by the LAD (52%) and the left circumflex artery (LCx; 40%). Moreover, in 37/44 (84%) twins only proximal lesions were reported, while distal lesions were reported in 7/44 (16%) twins with available data.

Of the 45 twins with available data, 25 (56%) underwent percutaneous coronary intervention (PCI) and 14 (31%) underwent coronary artery bypass graft surgery (CABG). Four twins (9%) had a PCI at first and then CABG, whereas two twins did not undergo revascularization, because of sudden cardiac arrest. The detailed procedural characteristics of the studied twin pairs are presented in Table 2.

### 3.5. Investigation of CAD-Related Mutations

Specific disease-causing mutation were investigated in five case reports (with 6 twin pairs) and four of them identified the following mutations:

1. Erythrocyte, leucocyte and chromosome polymorphisms (C and Q banding) [16];
2. Apolipoprotein E genotypes [21];
3. HLA [18];
4. LDLR c.1060+10G>A (rs12710260) mutation, heterozygous for the LDLR c.542C>T (rs557344672) mutations, homozygous for the c.1060+7T>C (rs2738442) and c.1586+53A>G (rs1569372) mutations in the LDLR gene and homozygous for the c.4265A>T (rs568413) mutations in the APOB gene [34].

### 3.6. Methodological Quality Assessment

The evaluation of the included studies according to their methodological quality indicated that most of them were of fair or good quality, as summarized in Supplementary Table S2. Specifically, 22 studies were rated with 5 stars, and 3 studies with 3 or 4 stars.

## 4. Discussion

The main findings of this systematic review and meta-summary of cases of concomitant CAD in identical twins are the following: (i) a total of 62 (31 pairs) identical twins suffered concomitantly from CAD (and 81% of them presented almost simultaneously with an ACS), (ii) all of the studied twins had at least one predisposing cardiovascular risk factor (most often family history of CAD), (iii) genetic analyses were only performed in six twin pairs and identified mutations in five of them, (iv) a total of 79% had concordant coronary anatomy, (v) a total of 77% had concordant lesions and most of those with concordant lesions shared predisposing risk factors, and (vi) a total of 84% of the twins had proximal lesions only.

The concomitant CAD presentation, the premature onset of CAD and the similar angiographical findings of the identical twins in this systematic review suggest that genetic factors play an important role in the development of CAD.

### 4.1. Twin Studies in CAD Susceptibility

CAD is a multifactorial disease, influenced by the interplay of environmental, epigenetic and genetic factors. Heritability is studied by comparing the extent of similarity between relatives in classic twin, twin-adoption, siblings/half-siblings and transgenerational family studies [38]. However, twin studies (unbiased by age effects) can reflect, quantify and disentangle the magnitude of genetic, (shared and unique) environmental components to a specific phenotype, by modeling the shared environment and accounting for maternal and early environmental factors [39]. Twins are usually exposed to a more similar family environment compared with non-twin siblings, and may share environmental variables and socioeconomic status even in adulthood, which can contribute to the expression of complex traits [40,41].

### 4.2. Genome

The genetic basis of CAD continues to be investigated; significant observations have been made based on the classic twin design, a compelling natural experiment over the course of the last century. The seminal Swedish twin registry [42] followed more than 20 thousand twins for 26 years, and reported that the genetic susceptibility to CAD-related death is mostly observed at a younger age and becomes less pronounced at an older age [16,43]. This is in line with the findings of our meta-summary of cases since the mean age of presentation was 45 years, whereas CAD prevalence in the general population is age-dependent being approximately 1%, 7% and 20% in age groups 18 to 44 years, 45 to 64 years and more than 65 years, respectively. The Danish Twin Concordance Study identified a higher concordance of CAD occurrence in identical than fraternal twins [16]. The twin design has shown that genetic factors exert a major effect on plasma concentrations of hemostatic proteins, causal lipoproteins or less-known atherothrombotic modulators [44,45]. Genetic factors may interact with physiological functions not only at one point in time but also over a period of time (interacting with their rates of change), which may in turn affect whether and when CAD occurs [8].

The BUDAPEST-GLOBAL (Burden of Atherosclerotic Plaques Study in Twins-Genetic Loci and the Burden of Atherosclerotic Lesions) classic twin study analyzed twin pairs without known CAD for the identification of specific genetic loci associated with atherosclerotic burden [46]. The investigators showed that calcified plaque volume is substantially determined by genetics whereas noncalcified plaque volume is mainly influenced by shared environmental factors [47,48,49,50,51]. These findings indicate that the early development of coronary plaques (usually noncalcified) is mainly influenced by environmental factors, in contrast to plaque calcification which is more dependent on genetics, occurs in later stages and might be less prone to cause adverse events [52]. This could correlate with our findings since most twin pairs shared environmental risk factors and had premature CAD (mean age of 45 years). Unfortunately, the nature of their coronary atherosclerotic plaques was not available.

Various CAD locations have different degrees of heritability. Left main or proximal disease has a high heritability pattern, whereas distal disease might be less influenced by genetic factors [53]. Environmental modulators or local hemodynamic factors (arterial shear stress alterations or coronary remodeling) may account for the dissimilar angiographic appearance of CAD [54,55,56]. The 20% discordant coronary anatomy (as documented in 5/24 studies with available data in our meta-summary) implies the involvement of non-hereditary factors in the modulation of coronary anatomy (dominant coronary vessel) leading to variability in the coronary blood supply in monozygotic twins.

Nevertheless, the ‘case of missing heritability’ is still open concerning the small number of susceptibility loci reproducibly identified by GWAS and their relatively small effects explaining only a small proportion of familial clustering. This shortcoming could be linked with the effect of rare or structural variants not captured by GWAS or with the existence of—so far unknown—gene–gene (epistasis) and gene–environment interactions and epi- or meta-genetic modifications.

### 4.3. Exposome

Exposome reflects the harmful biochemical and metabolic changes that occur in our body owing to the totality of different environmental exposures throughout the life course [57]. Since genetic variation explains only a modest proportion of the CAD risk [58], a large part of the disease burden should be attributed to environmental stressors and the gene–environment interplay (“genetics load the gun but environment pulls the trigger”). Our meta-summary suggests that most twins (with identical lesions) shared at least one environmental risk factor; this might be relevant with the common environmental factors shared by identical twin pairs during their (early) lifetime (same early childhood, education, location, dietary and exercise habits, similar socioeconomic status even in adulthood) [46,59].

The external environment might affect CAD risk in three broad domains: natural (air, water and land pollution), built (neighborhood level conditions, access to a healthy food environment) and social (psychosocial stress, demographic, economic, and political circumstances) [57]. These harmful exposures can increase the CAD risk synergistically with age, genetic predisposition and pre-existing risk factors. Hence, the exposome provides a framework for understanding how exposure pathways at different stages of life are involved in increasing the risk of CAD in identical twins with a discordant phenotype.

Even established environmental risk factors such as dyslipidemia, diabetes and physical activity are largely influenced by genetic mechanisms [60]; thus, it is not easy to accurately measure the amount of non-genetic variation that is due to environmental factors. Moreover, different environments in identical twins raised apart have not been associated with a higher degree of phenotypic discordance when compared with identical twins raised together [61]. These observations do not support the interpretation that the remaining (“non-genetic”) phenotypic variation in CAD phenotype of discordant identical twins can be safely attributed to environmental stressors.

### 4.4. Epigenome: “The Third Component”

The general conclusion drawn from the previous paragraphs is that remaining phenotypic discordance may occur in the absence of either genetic background differences or identifiable environmental variation. Epigenetics (i.e., unique DNA and chromatin modifications regulating the expression of similar genetic codes in different environmental conditions) could explain the missing heritability from GWAS studies as well as the divergence in gene expression between identical twins in the absence of observable environmental differences [61,62].

With the advance of the sequencing technologies, it is currently possible to analyze genome-wide epigenetic profiles at a relatively low cost [62]. Thereby, the dysregulation of epigenetics (e.g., altered patterns of miRNA expression and DNA methylation) have been linked with CAD/AMI occurrence, maybe as the mediators between social exposure and the underlying genome by altering stress and inflammation-related biological pathways [63,64,65,66,67,68].

The possibility that environmentally induced epigenetic changes may be transmitted across generations could theoretically account for a significant proportion of the missing phenotypic heritability [69]. Twin studies can also inform the role of intrauterine (e.g., parental diet and smoking, xenobiotics exposure, twin pair chorionicity, birthweight) and postnatal environmental factors for the establishment, maintenance and functional consequences of human epigenome variation [70]. Longitudinal epigenetic twin studies have demonstrated an “epigenetic drift” phenomenon, meaning that epigenetic changes may occur over time, even between birth and 18 months of age due to non-shared stochastic and environmental factors [71,72,73]. Different epigenetic profiles have been also identified in identical twin pairs despite deriving from a single zygotic epigenome. Hence, identical twins aged 45 years could have a considerable within-pair variability, and this could be even greater if the twins had divergent lifestyles in their adulthood [69].

Since monozygotic gene expression discordance levels are commonly above experimental noise, the ongoing EpiTwin study (http://www.epitrain.eu, accessed on 1 August 2023) will allow us to obtain a deeper understanding of the functional impact of epigenetic modifications on CAD through whole genome epigenetic profiling of phenotypically discordant identical twins. Unfortunately, no relevant data on epigenetics were available from our meta-summary of CAD cases in identical twins.

### 4.5. Metagenome

Another emerging and promising -omics field, in which discordant monozygotic twin studies could be an invaluable research tool, is metagenetics, the study of the metagenome (i.e., combined genes of human host with trillions of pathogen viruses or site-specific microbial communities). The interaction between microbiome and the host immune system contributes to the pathogenesis of inflammatory diseases in individuals carrying specific genetic variants. Hence, combining metagenetics with the EpiTwin study design will allow us to better understand the link between the metagenome and the multifactorial CAD onset.

### 4.6. Future Perspectives

With the ultimate goal of unraveling the multifactorial nature of CAD, monozygotic discordant twin studies combined with novel sequencing technologies are expected to provide important insights into the pathogenic role of epigenetic modifications and metagenetic interactions (gene–environment relationships). Given that epigenetic coding will be orders of magnitude more complex than genetic coding, the future focus of twin-based genomic and epigenomic analyses will fall on high throughput nucleotide sequencing, which would allow for the detection of (age-dependent) epigenetic variations at every site in every gene. With multiple ongoing international collaborative projects, epigenetics are projected to bolster our understanding and treatment of cardiovascular disorders. Understanding how epigenetics contribute to CAD will help develop future therapies and novel diagnostic tools.

Till then, our findings emphasize the importance of early lifestyle interventions and primary screening in asymptomatic twin siblings of CAD patients, as the likelihood of uncovering identical CAD lesions is high. Non-invasive imaging techniques or diagnostic panels of biomarkers with high specificity are warranted for routine cardiac diagnostics in asymptomatic twins at risk of CAD.

### 4.7. Limitations of Our Meta-Summary

Our study has limitations. First, the sample size of identical twin pairs was small (n = 31) and, hence, we cannot draw definite conclusions. Second, several of the included case reports did not provide some of the data examined in this meta-summary (e.g., CAD symptoms, risk factors, laboratory or angiographic data). The case reports included in our meta-summary provided limited evidence on disease-causing mutations (only 6/31 twin pairs were subject to GWAS analysis as reported). Future twin studies undergoing (epi-)genomic analyses for concomitant CAD presentation are warranted to shed light on non-investigated genetic and epigenetic polymorphisms.

## 5. Conclusions

In summary, monozygotic twins can present simultaneously with CAD and have high concordance in coronary anatomy and CAD lesions. In this meta-summary of 31 cases, most of the studied twins shared predisposing risk factors (most often family history of CAD), and proximal lesions were most frequently observed. We can conclude that asymptomatic twins of symptomatic counterparts require aggressive assessment and prevention for CAD. Over the next decade, a full characterization of human genomic, epigenomic and transcriptomic data may further clarify the proportion of genetic, epigenetic or environmental components to (premature) CAD development.

## Figures and Tables

**Table 1:** Baseline characteristics of the studied twin pairs (mean ± SD or n (%)).

**Variable (N)**		31 pairs or 62 twins
**Age, years**		45 ± 12
**Males (/31 pairs)**		25 (81)
**Presenting Condition (/62 twins)**		
Unstable Angina		27 (43)
AMI		25 (40)
Positive Stress Test		2 (3)
Low blood pressure		1 (2)
Cardiac Arrest		1 (2)
None		6 (10)
**Presenting symptoms/signs (/62 twins)**		
Chest pain		38 (86)
Dyspnea/Respiratory distress		6 (14)
4th heart sound		2 (5)
Edema		1 (2)
Nausea		2 (5)
Hyperhidrosis		3 (7)
None		4 (9)
Missing data		18 (29)
**Cardiovascular risk factors (/62 twins)**		
Current smoking		30 (48)
Past smoking		2 (3)
Dyslipidemia		37 (60)
Hypertension		21 (34)
Obesity		7 (11)
Diabetes/prediabetes		10 (16)
Familial Hypercholesterolemia		2 (3)
Alcohol		1 (2)
Cocaine		1 (2)
Hypothyroidism		2 (3)
None		1 (2)
**Family History of CAD (/31 pairs)**		
Yes		16 (80)
Missing data		11 (35)
**Laboratory workup (mg/dL)**		
Total cholesterol	8/31 pairs	256 ± 44
Low density lipoprotein	5/31 pars	134 ± 76
High density lipoprotein	2/31 pairs	39 ± 11
Triglycerides	19/62 twins	189 ± 108
Lipoprotein(a)	7/62 twins	207 ± 73

AMI, acute myocardial infarction; CAD, coronary artery disease.

**Table 2:** Procedural characteristics of the studied twin pairs (mean ± SD or n (%)).

**Variable (N)**	31 pairs or 62 twins
**Anatomy Concordance (/31 pairs)**	
Yes	19 (79)
Missing data	7 (23)
**Concordance of Lesions (/31 pairs)**	24 (77)
**Diseased vessel (/62 twins)**	
Right coronary artery	33 (55)
Left main coronary artery	4 (7)
Left anterior descending artery	31 (52)
Left circumflex artery	24 (40)
Obtuse marginal artery	2 (3)
Posterior left ventricular artery	2 (3)
Posterior descending artery	3 (5)
Diagonal branches of the left anterior descending artery	1 (2)
Three-vessel disease	2 (3)
Missing data	3 (5)
**Site of occlusion (/62 twins)**	
Proximal	37 (84)
Distal	1 (2)
Both distal and proximal	6 (14)
Missing data	18 (29)
**Revascularization Procedures (/62 twins)**	
PCI	25 (56)
CABG	14 (31)
PCI and CABG	4 (9)
None (died)	2 (4)
Missing data	17 (28)

CABG, coronary artery bypass graft surgery; PCI, percutaneous coronary intervention.

## Data Availability

Data are available upon reasonable request from the corresponding author of the study.

## Supplementary Materials

The following supporting information can be downloaded at: https://www.mdpi.com/article/10.3390/jcm12175742/s1, Figure S1: Cardiovascular disease-associated single nucleotide polymorphisms investigated in the reported case of monozygotic twins; Table S1: basic information regarding included studies.; Table S2: methodological quality of included studies assessing and grading certain domains.
