# Supplementary material for: Concomitant Coronary Artery Disease in Identical Twins: Case Report and Systematic Literature Review
Source: J Clin Med. 2023 Sep 3;12(17):5742. doi: 10.3390/jcm12175742 (PMC10489011; doi:10.3390/jcm12175742)
Supplement: Supplementary file 1 [file jcm-12-05742-s001.zip › jcm-2596258-supplementary.pdf]

Supplementary Table S1. Basic information regarding the included studies.

**Study**	**Country**	**Gender**	**Age at onset** *(twin 1/ twin 2)*	**Presenting Symptoms/Signs** *(twin 1/ twin 2)*	**Presenting Condition** *(twin 1/ twin 2)*	**Risk Factors** *(twin 1/ twin 2)*	**Treatment** *(twin 1/ twin 2)*
Giknis et al. 1965 [1]	USA	M	20/20	Chest pain, Nausea, Hyperhidrosis/ Chest pain, Nausea, Hyperhidrosis, Dyspnea	Myocardial infarction/ Myocardial infarction	NR/ Smoking, Dyslipidemia	NR/ NR
Sidd et al. 1966 [2]	USA	M	43/42	Chest pain/ Chest pain	Unstable angina/ Unstable angina	Smoking, Dyslipidemia/ Smoking, Dyslipidemia	NR/ NR
Kreulen et al. 1975 [3]	USA	M	30/30	Chest pain/ Chest pain, Diaphoresis	Unstable angina/ Unstable angina	Dyslipidemia, Systolic hypertension/ Dyslipidemia, Systolic hypertension	CABG/ CABG
Holmes et al. 1981 [4]	USA	M	32/33	NR	Unstable angina/ Myocardial infarction	Smoking, Dyslipidemia/ Smoking, Dyslipidemia	NR/ CABG
		M	48/47	4th heart sound, Systolic ejection murmur/ 4th heart sound	Unstable angina/ Unstable angina	Hypertension, Dyslipidemia/ Hypertension, Dyslipidemia, Smoking	NR/ CABG
Herrington et al. 1987 [5]	USA	M	46/47	NR	Myocardial infarction/ Myocardial infarction	Smoking/ Smoking	NR/ NR
		M	44/46	NR	Unstable angina / Unstable angina	Smoking, Diabetes mellitus, Systolic hypertension, Dyslipidemia/ Smoking, Diabetes mellitus, Systolic hypertension, Dyslipidemia	NR/ NR
Kanda et al. 1991 [6]	Japan	M	37/-	Respiratory distress/ none	Myocardial infarction/ Unstable angina	Diabetes mellitus, Smoking, Obesity/ Diabetes mellitus	NO (died)/ NR
Samuels et al. 1999 [7]	USA	F	66/68	Retrosternal chest discomfort radiating to left axilla/ none	Unstable angina / Angina	Dyslipidemia/ Dyslipidemia	CABG/ CABG

Frings et al. 2000 [8]	Germany	M	29/29	NR	Myocardial infarction/ Positive stress test	Dyslipidemia/ Dyslipidemia	CABG/ PCI & CABG
		F	49/51	NR	Unstable angina/ Unstable angina	Dyslipidemia, Hypertension, Diabetes mellitus/ Dyslipidemia, Hypertension	PCI & CABG/ PCI & CABG
		M	41/47	NR	Myocardial infarction/ Angina	Smoking, Obesity, Hypertension/ Smoking, Dyslipidemia	PCI/ PCI & CABG
Serdar et al. 2000 [9]	Turkey	M	43/43	NR	Myocardial infarction/ Myocardial infarction	Smoking/ Smoking	CABG/ CABG
Kaluza et al. 2000 [10]	USA	M	55/55	NR	Unstable angina/ Unstable angina	Smoking, Dyslipidemia/ Smoking, Dyslipidemia	PCI/ PCI
Nathoe et al. 2002 [11]	Netherland s	F	58/58	NR	Unstable angina/ Unstable angina	Dyslipidemia, Smoking, Hypertension, Obesity/ Obesity	PCI/ PCI
		M	39/34	NR	Unstable angina/ Myocardial infarction	Smoking/ Smoking	CABG/ CABG
Cokkinos et al. 2006 [12]	Greece	F	65/65	NR	Unstable angina / Unstable angina	Dyslipidemia, Diabetes mellitus/ Dyslipidemia, Diabetes mellitus, Hypertension	PCI/ PCI
Gullu et al. 2007 [13]	Turkey	M	62/62	NR/ non-specific angina symptoms	Unstable angina / Unstable angina	Dyslipidemia, Obesity, Smoking/ Obesity	CABG/ CABG
Segura et al. 2007 [14]	Spain	M	49/49	NR	Unstable angina/ Unstable angina	Dyslipidemia, Smoking, Hypertension/ Dyslipidemia, Smoking, Hypertension	PCI/ PCI
Turley et al. 2008 [15]	UK	M	46/46	Chest discomfort/ Chest discomfort	Unstable angina / Stable angina	Dyslipidemia, Hypertension, Smoking, Obesity/ Dyslipidemia, Hypertension	PCI/ PCI
Douglas et al. 2009 [16]	UK	M	47/42	NR	Myocardial infarction/ Myocardial infarction	Dyslipidemia, Smoking, Hypertension/	NR/ NR

						Dyslipidemia, Hypertension	
Kashish et al. 2015 [17]	USA	F	59/60	Chest pain/ NR	Unstable angina/ Cardiac arrest	Dyslipidemia, Smoking, Hypertension/ NR	NR/ NR (died)
Papanikolaou et al. 2017 [18]	Greece	F	45/45	Atypical chest pain/ Precordial pain at rest	Myocardial infarction / Unstable angina	Hypertension/ Smoking	PCI/ PCI
Grabowicz et al. 2019 [19]	Poland	M	53/53	NR/ Dyspnea upon exertion	Myocardial infarction/ ECG treadmill test	Dyslipidemia/ Dyslipidemia	PCI/ PCI
Clement et al. 2020 [20]	Paris	M	46/47	Chest oppression, Left arm discomfort/ Chest pain, Dyspnea	Myocardial infarction/ Myocardial infarction	Smoking/ Smoking	PCI/ PCI
Kern et al. 2020 [21]	Poland	M	53/54	NR	Myocardial infarction/ Low blood pressure	Dyslipidemia, Hypothyroidism/ Dyslipidemia, Hypothyroidism	PCI/ PCI
Kayıkçıoğlu et al. 2020 [22]	Turkey	M	33/32	NR	Myocardial infarction/ Myocardial infarction	Ex-smoker, Familial Dyslipidemia / Ex-smoker, Familial Dyslipidemia	NR/ PCI
Smith et al. 2021 [23]	USA	M	42/40	Edema, Dyspnea/ NR	Unstable angina / Myocardial infarction	Diabetes mellitus, Alcohol, Cocaine, Hypertension/ NR	CABG/ NR
Singh et al. 2022 [24]	India	M	36/36	Chest pain/ Dyspnea on exertion	Unstable angina / Stable angina	Dyslipidemia/ Dyslipidemia	PCI/ PCI
Kameel et al. 2023 [25]	USA	M	54/54	Chest pain/ Chest pain	Unstable angina / Myocardial infarction	Pre-Diabetes/ Pre-Diabetes	NO/ PCI
Our case:	Greece	M	46/46	Chest pain/ Chest pain	Myocardial infarction/ Unstable angina	Smoking/ Smoking	PCI/ PCI

CABG, coronary artery bypass graft surgery; F, female; M, male; NR, not reported; PCI, percutaneous coronary intervention

Supplementary Table S2.
Methodological quality of included studies assessing and grading certain domains

Study	Selection	Ascertainment	Causality	Reporting	Total
Giknis et al. 1965 [1]	☆	☆☆	☆	☆	5/5
Sidd et al. 1966 [2]	☆	☆☆	☆	☆	5/5
Kreulen et al. 1975 [3]	☆	☆☆	☆	☆	5/5
Holmes et al. 1981 [4]	☆	☆☆	☆	☆	5/5
Herrington et al. 1985[5]	☆	☆☆	☆	☆	5/5
Kanda et al. 1991[6]	☆	☆☆	☆	☆	5/5
Samuels et al. 1999[7]	☆	☆☆	☆	☆	5/5
Frings et al. 2000[8]	☆	☆☆	☆	☆	5/5
Serdar et al. 2000[9]	☆	☆		☆	3/5
Kaluza et al. 2000[10]	☆	☆	☆		3/5
Nathoe et al. 2002[11]	☆	☆☆	☆	☆	5/5
Cokkinos et al. 2006[12]	☆	☆☆	☆	☆	5/5
Gullu et al. 2007[13]	☆	☆☆	☆	☆	5/5
Segura et al. 2007[14]	☆	☆☆	☆	☆	5/5
Turley et al. 2008[15]	☆	☆☆	☆	☆	5/5
Douglas et al. 2009[16]	☆	☆☆	☆	☆	5/5
Kashish et al. 2015[17]	☆	☆☆	☆	☆	5/5
Papanikolaou et al. 2017 [18]	☆	☆☆	☆	☆	5/5
Grabowicz et al. 2019[19]	☆	☆☆	☆	☆	5/5
Clement et al. 2020[20]	☆	☆☆	☆	☆	5/5
Kern et al. 2020[21]	☆	☆☆	☆		4/5

Kayıkçıoğlu et al. 2020[22]	☆	☆☆	☆	☆	5/5
Smith et al. 2021[23]	☆	☆☆	☆	☆	5/5
Singh et al. 2022[24]	☆	☆☆	☆	☆	5/5
Kameel et al. 2023[25]	☆	☆☆	☆	☆	5/5

The methodological quality of each study was assessed after rating each study according to the following questions:
Selection: 1. Does the patient(s) represent(s) the whole experience of the investigator (centre) or is the selection method unclear to the extent that other patients with similar presentation may not have been reported? (yes/no)
Ascertainment: 2. Was the exposure adequately ascertained? (yes/no); 3. Was the outcome adequately ascertained? (yes/no)
Causality: 4. Was follow-up long enough for outcomes to occur? (yes/no)
Reporting: 5. Is the case(s) described with sufficient details to allow other investigators to replicate the research or to allow practitioners make inferences related to their own practice? (yes/no).

References

1. GIKNIS FL, HOLT DE, WHITEMAN HW, SINGH MD, BENCHIMOL A, DIMOND EG. MYOCARDIAL INFARCTION IN TWENTY-YEAR-OLD IDENTICAL TWINS. Am J Cardiol [Internet]. 1965 Jul 1 [cited 2023 May 23];16(1):122–6. Available from: http://www.ncbi.nlm.nih.gov/pubmed/14314197
2. Sidd JJ, Sasahara AA, Littmann D. Coronary-Artery Disease in Identical Twins. New England Journal of Medicine [Internet]. 1966 Jan 13;274(2):55–60. Available from: https://doi.org/10.1056/NEJM196601132740201
3. Kreulen TH, Cohn PF, Gorlin R. Catheterization and Cardiovascular Diagnosis 1 :91-96 (1975) Case Report PREMATURE CORONARY ARTERY DISEASE IN IDENTICAL MALE TWINS STUDIED BY SELECTIVE CORONARY ARTERIOGRAPHY. 1975.
4. Holmes Jr DR, Kennel AJ, Smith HC, Gordon H, Moore B. Coronary artery disease in twins [Internet]. Vol. 45, Br Heart J. 1981. Available from: http://heart.bmj.com/
5. Herrington DM, Pearson TA. Clinical and Angiographic Similarities in Twins with Coronary Artery Disease. 1985.
6. Kanda T, Ohshima S, Yuasa K, Toyama T, Takase SI, Maebashi P, et al. Painless Myocardial Infarction in Identical Diabetic Twins. Vol. 30, Jpn J Med. 1991.
7. Samuels LE, Samuels FS, Thomas MP, Morris RJ, Wechsler AS. Coronary artery disease in identical twins. Ann Thorac Surg [Internet]. 1999 Aug 1 [cited 2023 May 23];68(2):594–600. Available from: https://linkinghub.elsevier.com/retrieve/pii/S0003497599006293
8. Frings AM, Mayer B, Böcker W, Hengstenberg C, Willemsen D, Riegger GAJ, et al. Comparative coronary anatomy in six twin pairs with coronary artery disease. Heart. 2000;83(1):47–50.
9. Ener S. Coronary artery disease in identical twins. Ann Thorac Surg [Internet]. 2000 Aug 1 [cited 2023 May 23];70(2):692. Available from: https://linkinghub.elsevier.com/retrieve/pii/S0003497500016519

10. Kaluźa G, Abukhalil JM, Raizner AE. Identical atherosclerotic lesions in identical twins. Circulation. 2000;101(4).
11. Nathoe HMW, Stella PR, Eefting FD, de Jaegere PPT. Angiographic findings in monozygotic twins with coronary artery disease. Am J Cardiol [Internet]. 2002 Apr 15 [cited 2023 May 23];89(8):1006–9. Available from: http://www.ncbi.nlm.nih.gov/pubmed/11950451
12. Cokkinos P, Sbarouni E, Kremastinos DT. Coronary angioplasty in identical twins. Int J Cardiol [Internet]. 2006 Mar 8 [cited 2023 May 23];107(3):434–5. Available from: http://www.ncbi.nlm.nih.gov/pubmed/16503274
13. Gullu AU, Kizilay M, Ates M, Akcar M. The comparison of angiographic lesions and clinical outcomes in identical twins. Interact Cardiovasc Thorac Surg. 2007 Aug;6(4):575–6.
14. Segura L, Moreno R, Macaya C. Coronary Artery Disease and Percutaneous Coronary Intervention in a Set of Twins. Revista Española de Cardiología (English Edition) [Internet]. 2007;60(1):86–7. Available from: https://www.revespcardiol.org/en-coronary-artery-disease-percutaneous-coronary-articulo-13098661
15. Turley AJ, Chen V, Hall JA. Simultaneous presentation of coronary heart disease in identical twins. Postgrad Med J [Internet]. 2008 Feb 1;84(988):100–2. Available from: https://doi.org/10.1136/pgmj.2007.064493
16. Hanna D. CORONARY ARTERY DISEASE: ANATOMY AND PRESENTATION IN IDENTICAL TWINS IN IDENTICAL . Ulster Med J. 2009 Sep;
17. Goel K, Tweet M, Olson TM, Maleszewski JJ, Gulati R, Hayes SN. Familial Spontaneous Coronary Artery Dissection: Evidence for Genetic Susceptibility. JAMA Intern Med [Internet]. 2015 May 1;175(5):821–6. Available from: https://doi.org/10.1001/jamainternmed.2014.8307
18. Papanikolaou J et al. Twin Hearts in Identical Twins. JOURNAL OF Invasive Cardiology. 2017 Jul;
19. Grabowicz W, Masiarek K, Warchoł I, Górnik T, Lubiński A. Déjà vu: Coronary artery disease in monozygotic twins. Vol. 77, Kardiologia Polska. Medycyna Praktyczna Cholerzyn; 2019. p. 886–7.
20. Clement A, Picard F, Varenne O. Myocardial infarction in monozygotic twins. Vol. 13, BMJ Case Reports. BMJ Publishing Group; 2020.
21. Kern A, Bojko K, Sienkiewicz E, Zarzecki A, Bil J. Non-st-elevation acute coronary syndrome due to a totally occluded coronary artery: a history of two twin brothers. Wiad Lek. 2020;73(1):201–2.
22. Kayikçioğlu M, Uzun HG, Vardarli AT, Tokgözoğlu L. Monozygotic twins with familial hypercholesterolemia and high lipoprotein(a) levels leading to identical cardiovascular outcomes: Case report and review of the literature. Turk Kardiyoloji Dernegi Arsivi. 2020 Jul 1;48(5):531–8.
23. Smith MC, Baker JR, Gleaves E, Singh A, Kazimuddin M. Twinning: Coronary Artery Disease in Monozygotic Twins. Cureus [Internet]. 2021;13(7):e16139. Available from: http://dx.doi.org/10.7759/cureus.16139
24. Singh AP, Raj A, Bankar B, Nath RK. Enigma of Twins: Identical Presentation and Angiographic Lesion in Monozygotic Twins. J Saudi Heart Assoc. 2022;34(1):6–10.

25. Kassab K, Gupta V. Simultaneous ST-elevation myocardial infarction in monozygotic twins: a case report of entangled twins. Eur Heart J Case Rep. 2023 Feb 3;7(2).

Supplementary Figure S1. Cardiovascular disease-associated single nucleotide polymorphisms investigated in the current case of monozygotic twins

AARS2, ABCA1, ABCC6, ABCC8, ABCC9, ABCG5, ABCG8, ABL1, ACAD9, ACADVL, ACTA1, ACTA2, ACTB, ACTC1, ACTG1, ACTN2, ACVR1, ACVR2B, ACVRL1, ADAMTS10, ADAMTS17, ADAMTS2, ADAMTSL4, AFF4, AGK, AGL, AGPAT2, AKAP9, ALDH18A1, ALMS1, ALPK3, AMMECR1, ANK2, ANKS6, ANO5, APOA1, APOA5, APOB, APOC2, APOC3, APOE, AQP1, ARHGAP31, ARID1A, ARID1B, ARMC4, ATP13A3, ATP6V0A2, ATP7A, ATPAF2, B3GAT3, B4GALT7, BAG3, BCOR, BGN, BMPR1B, BMPR2, BRAF, C12ORF57, C21ORF59, CACNA1C, CACNB2, CALM1, CALM2, CALM3, CALR3, CAPN3, CASQ2, CASZ1, CAV1, CAV3, CBL, CBS, CCDC103, CCDC114, CCDC151, CCDC39, CCDC40, CCNK, CDC42, CDH2, CDK13, CDK9, CFAP53, CHD4, CHD7, CHRM2, CHST14, COL11A1, COL11A2, COL1A1, COL1A2, COL2A1, COL3A1, COL4A5, COL5A1, COL5A2, COLGALT1, COX15, CPT2, CREB3L3, CREBBP, CRELD1, CRYAB, CSRP3, CTC1, CTNNA3, DBH, DES, DHCR7, DLG4, DLL4, DMD, DNAAF1, DNAAF2, DNAAF3, DNAAF5, DNAH11, DNAH5, DNAI1, DNAI2, DNAJC19, DNAL1, DOCK6, DOLK, DPM3, DSC2, DSE, DSG2, DSP, DTNA, DYSF, DYX1C1, EEF1A2, EFEMP2, EFTUD2, EHMT1, EIF2AK4, ELAC2, ELN, EMD, ENG, ENPP1, EOGT, EP300, EPG5, EPHB4, ETFA, ETFB, ETFDH, EVC, EVC2, FAH, FBLN5, FBN1, FBN2, FBXL4, FBXO32, FGD1, FHL1, FHOD3, FKBP14, FKRP, FKTN, FLNA, FLNC, FOXC1, FOXD4, FOXE3, FOXF1, FOXH1, FOXP1, FOXRED1, FXN, GAA, GATA4, GATA5, GATA6, GATAD1, GATC, GBE1, GDF1, GDF2, GFM1, GJA1, GJA5, GLA, GLB1, GMPPB, GPC3, GPIHBP1, GSK3B, GTPBP3, GUSB, HADHA, HAND1, HAND2, HCN4, HDAC8, HFE, HNRNPK, HOXA1, HRAS, IDUA, ILK, INVS, ISPD, JAG1, JPH2, JUP, KAT6B, KCNA5, KCNE1, KCNE2, KCNH2, KCNJ2, KCNJ5, KCNK3, KCNQ1, KDM6A, KLF2, KLHL24, KMT2D, KRAS, KYNU, LAMA2, LAMP2, LARGE, LDB3, LDLR, LDLRAP1, LEFTY2, LEMD2, LIPA, LMF1, LMNA, LMOD2, LOX, LPL, LRRC10, LRRC6, LZTR1, MAP2K1, MAP2K2, MAP3K8, MAT2A, MED12, MED13L, MEIS2, MFAP5, MIPEP, MLYCD, MMP21, MRAS, MRPL3, MRPL44, MRPS22, MYBPC3, MYBPHL, MYCN, MYH11, MYH6, MYH7, MYL2, MYL3, MYL4, MYLK, MYO18B, MYOT, MYPN, MYRF, NAA15, NDUFAF2, NEXN, NF1, NF2, NFU1, NIPBL, NKX2-5, NKX2-6, NODAL, NONO, NOS1AP, NOTCH1, NOTCH2, NOTCH3, NR2F2, NRAP, NRAS, NSD1, NSUN2, NUP155, PARS2, PCCA, PCCB, PCSK9, PIH1D3, PITRM1, PITX2, PKD1L1, PKP2, PLEC, PLEKHM2, PLN, PLOD1, PNPLA2, POMT1, PPA2, PPCS, PPP1CB, PRDM16, PRDM6, PRKAG2, PRKD1, PRKG1, PTPN11, PUF60, PYCR1, QRSL1, RAB23, RAF1, RASA1, RASA2, RBCK1, RBM10, RBM20, RERE, RIT1, RMND1, RRAS, RYR2, SALL4, SARS2, SASH1, SCN10A, SCN1B, SCN3B, SCN5A, SCNN1B, SCNN1G, SCO1, SCO2, SDHA, SELENON, SGCA, SGCB, SGCD, SGCG, SHOC2, SKI, SLC22A5, SLC25A20, SLC25A3, SLC25A4, SLC2A10, SLC39A13, SMAD2, SMAD3, SMAD4, SMAD6, SMAD9, SMARCB1, SMC1A, SMC3, SMCHD1, SOS1, SOS2, SOX17, SPAG1, SPEG, SPRED1, STAG2, STAMBP, STRA6, SYNGAP1, TAB2, TAZ, TBX1, TBX20, TBX4, TBX5, TCAP, TECRL, TFAP2B, TGDS, TGFB2, TGFB3, TGFBR1, TGFBR2, TLL1, TMEM43, TMEM70, TMEM94, TNNC1, TNNI3, TNNI3K, TNNT2, TOR1AIP1, TPM1, TRDN, TRIM32, TRPM4, TSFM, TTC25, TTN, TTR, UPF3B, VARS2, VCAN, VCL, VCP, VPS13A, XK, ZDHHC9, ZEB2, ZFPM2, ZIC3, ZMYND10, ZNF469
